# Annual Estimation of Seasonal Influenza Burden in 6 South American Countries: A Retrospective Analysis of SARInet Surveillance Data to Inform Policies

**DOI:** 10.1093/infdis/jiaf037

**Published:** 2025-03-10

**Authors:** Miguel Angel Descalzo, Francisco José de Paula Júnior, Natalia Vergara Mallegas, Elena Penayo, Carla Voto, Natalia Goñi, Alfredo Bruno, Walquiria Aparecida Ferreira da Almeida, Greice Madeleine Ikeda do Carmo, María Fernanda Olivares Barraza, Rodrigo Fasce, Jorge Pacheco, Cynthia Vázquez, Marta Von Horoch, Silvia Battaglia, Carlos Giovacchini, Elsa Baumeister, Adrián Santoro, María Pía Buyayisqui, Miguel Alegretti, Mónica Patricia Escobar Naranjo, Jorge H Jara, Francisco Nogareda, Ángel Rodríguez, Nelson Jose Alvis-Zakzuk, A Danielle Iuliano, Eduardo Azziz-Baumgartner, Stefano Tempia, Juliana Almeida Leite, Marc Rondy, Paula Couto

**Affiliations:** Department of Public Health Emergencies, Pan American Health Organization/World Health Organization, Washington, DC, USA; Ministry of Health, Brasilia, Brazil; Department of Epidemiology, Ministry of Health, Santiago, Chile; General Directorate of Health Surveillance, Ministry of Public Health and Social Welfare, Asunción, Paraguay; Surveillance Area, Directorate of Epidemiology, Ministry of Health, Buenos Aires, Argentina; Department of Health Surveillance, Ministry of Public Health, Montevideo, Uruguay; National Reference Center for Influenza and other Respiratory Viruses, National Institute of Public Health Research, Guayaquil, Ecuador; Universidad Agraria del Ecuador, Guayaquil, Ecuador; Ministry of Health, Brasilia, Brazil; Ministry of Health, Brasilia, Brazil; Department of Epidemiology, Ministry of Health, Santiago, Chile; Viral Diseases Department, Institute of Public Health, Santiago, Chile; Department of Epidemiology, Ministry of Health, Santiago, Chile; General Directorate of Health Surveillance, Ministry of Public Health and Social Welfare, Asunción, Paraguay; General Directorate of Health Surveillance, Ministry of Public Health and Social Welfare, Asunción, Paraguay; General Directorate of Health Surveillance, Ministry of Public Health and Social Welfare, Asunción, Paraguay; Surveillance Area, Directorate of Epidemiology, Ministry of Health, Buenos Aires, Argentina; Respiratory Viruses Service, National Reference Laboratory, Pan American Health Organization/World Health Organization National Influenza Center, INEI-ANLIS “Dr. Carlos G. Malbrán”, Buenos Aires, Argentina; Directorate of Statistics and Health Information, Ministry of Health, Buenos Aires, Argentina; Surveillance Area, Directorate of Epidemiology, Ministry of Health, Buenos Aires, Argentina; Department of Health Surveillance, Ministry of Public Health, Montevideo, Uruguay; National Directorate of Epidemiological Surveillance, Ministry of Public Health, Quito, Ecuador; Special Program Comprehensive Immunization, Pan American Health Organization/World Health Organization, Washington, DC, USA; Special Program Comprehensive Immunization, Pan American Health Organization/World Health Organization, Washington, DC, USA; Department of Public Health Emergencies, Pan American Health Organization/World Health Organization, Washington, DC, USA; Department of Public Health Emergencies, Pan American Health Organization/World Health Organization, Washington, DC, USA; Influenza Division, National Center for Immunization and Respiratory Diseases, US Centers for Disease Control and Prevention, Atlanta, Georgia, USA; Influenza Division, National Center for Immunization and Respiratory Diseases, US Centers for Disease Control and Prevention, Atlanta, Georgia, USA; Global Influenza Program, World Health Organization, Geneva, Switzerland; Department of Public Health Emergencies, Pan American Health Organization/World Health Organization, Washington, DC, USA; Department of Public Health Emergencies, Pan American Health Organization/World Health Organization, Washington, DC, USA; Department of Public Health Emergencies, Pan American Health Organization/World Health Organization, Washington, DC, USA

**Keywords:** burden of disease, mild illness, hospitalizations, deaths, multiplier model

## Abstract

**Background:**

We estimate annual viral influenza-associated mild-to-moderate illness, hospitalizations, and deaths in 6 South American countries (Argentina, Brazil, Chile, Ecuador, Paraguay, and Uruguay) during the 2015–2019 influenza seasons as a first step in evaluating the full value of influenza vaccination in the subregion.

**Methods:**

We applied a multiplier method using monthly hospital discharge and vital statistics death records, influenza surveillance data, and population projections to estimate mild-to-moderate influenza-associated illness, hospitalizations, and deaths. We estimated the uncertainty bounds based on the 2.5th and 97.5th percentiles of the Monte Carlo simulated distributions for the number of cases and obtained the ranges from the minimum value of the 2.5th and the maximum value of the 97.5th percentile.

**Results:**

In selected countries with a total population of 307 million people, the yearly influenza-associated burden of disease ranged between 51 and 78 million mild-to-moderate influenza illnesses, between 323 379 and 490 049 hospitalizations, and between 22 662 and 46 971 deaths during the 2015–2019 influenza seasons.

**Conclusions:**

Each year, influenza is associated with millions of illnesses, hundreds of thousands of hospitalizations, and tens of thousands of deaths in 6 South American countries, affecting a significant portion of the population. Such findings can be used to estimate the number of illnesses averted through vaccination programs and the cost-benefit of influenza vaccines.

Estimates of viral influenza burden of disease (BoD) are useful to guide resources and policies for influenza epidemics and pandemic prevention and control [1, 2]. While challenging to accurately estimate, information about the BoD across the full spectrum of illness severity can guide comprehensive economic burden, vaccine impact, and cost-effectiveness evaluations [3]. Annual BoD estimates, combined with data about influenza vaccine effectiveness and coverage, can allow for rapid assessment of prevention and control measures [4]. In turn, such evaluations can help health authorities make evidence-based decisions and refine policies to optimize influenza mitigation.

In North America, influenza surveillance data have been used for years to estimate the BoD, but fewer data are published from Central and South America [5, 6]. Indeed, there is comparatively little information about the influenza BoD in Central and South America to guide investments in influenza mitigation. In the past, influenza BoD estimates from the Americas focused on hospitalization and associated deaths [7, 8], leaving gaps in understanding on BoD among those with milder but more common illnesses. To address this gap in knowledge, the Severe Acute Respiratory Infections network (SARInet) sought to leverage improved surveillance data and methods to estimate the influenza BoD in South America [9, 10].

In 2015, the World Health Organization (WHO) developed a manual for estimating the disease burden associated with seasonal influenza [11]. Since then, SARInet implemented regional and in-country trainings to enable countries to estimate influenza BoD using standardized approaches [7, 12–16]. As highlighted in the WHO manual [11], estimating the burden of mild to moderate nonhospitalized cases attributable to influenza is also important because of the large number of such cases occurring annually, their associated economic burden, and their adverse impact on healthcare systems and society [17]. Retrospective estimates of influenza BoD across multiple seasons enable health authorities to assess seasonal variation and optimize influenza prevention and control policies [4, 6]. In the current analysis, we estimated the annual burden of mild-to-moderate illness, hospitalizations, and deaths attributed to influenza in 6 South American countries during the 2015–2019 influenza seasons.

## METHODS

### Study Design and Data Sources

For this analysis, we obtained retrospective data from 6 countries that participate in the SARInet BoD working groups: Argentina, Brazil, Chile, Ecuador, Paraguay and Uruguay. The analysis included data from 1 January 2015 to 31 December 2019. We used monthly hospital discharge records and monthly deaths classified according to the *International Classification of Diseases, Tenth Revision* (*ICD-10*). Data about hospitalization first-listed discharge diagnoses were obtained from national electronic health information systems available through countries’ ministries of health. Data about first-listed cause of death were obtained from vital statistics departments or national institutes of statistics and census. We then identified all respiratory illness discharge diagnoses and cause of death (*ICD-10* codes J00–J99) for our analysis. When hospitalization data were incomplete for some jurisdictions, we produced estimates for jurisdictions with available data and then extrapolated these to the rest of a country based on population size. Influenza surveillance data were obtained from each country's national influenza centers [15, 16, 18, 19]. We used the monthly number of respiratory samples tested for influenza and the monthly number that tested positive for influenza virus, to attribute respiratory hospitalizations and deaths to influenza illness by age groups (ie, <5, 5–64, or ≥65 years) [10]. We used data from national institutes of statistics by age group to estimate the population at risk of influenza illness each year.

### Annual Estimates of Influenza-Associated Respiratory Hospitalizations and Deaths

To estimate the annual number of influenza-associated respiratory hospitalizations and deaths in each country, we used a multiplier method, which assumed that, every month, the number of all-respiratory hospitalizations or deaths attributable to influenza by age was proportional to the percentage of influenza-positive specimens identified at national influenza centers during same month. Methodologic analysis, calculations, and assumptions were similar to those in previous studies [7, 14, 15]. We first estimated the monthly percentage of virologic surveillance samples that tested positive for influenza by age group. Then, we estimated the monthly numbers of influenza-associated hospitalizations (or deaths) by multiplying the age-stratified number of respiratory hospitalizations (or deaths) in a month by the age-stratified influenza virus circulation in the same month. We then summed monthly age-stratified hospitalizations (or deaths) across each year to estimate the annual number of influenza-associated respiratory hospitalizations (or deaths). Surveillance data has been used only to determine the percentage of positive samples. Data on respiratory hospitalizations and deaths were used from national statistics to multiply by the percentage of influenza-positive samples. Thereafter, we used 100 000 Monte Carlo simulations to create a distribution of estimates by age group and country, assuming a Poisson distribution. We used the 2.5th and 97.5th percentiles of the simulated distributions by each age-specific group, year, and country to describe the uncertainty of our estimates, or the 95% credible intervals.

### Annual Estimates of Mild-to-Moderate Influenza Illness

We used a web tool built from a systematic literature review and meta-analysis to estimate mild-to-moderate influenza-associated illness from our estimated influenza-associated hospitalizations and/or deaths [20]. The tool was developed by the Johns Hopkins Center for Health Security Bloomberg School of Public Health, in partnership with WHO, and has been used previously [21]. We estimated the uncertainty bounds based on the 2.5th and 97.5th percentiles for influenza-associated respiratory hospitalizations and deaths.

### Statistical Analyses

We estimated incidence (per 100 000 population) of mild-to-moderate influenza illness, influenza-associated hospitalizations and influenza-associated deaths by age group and country. Incidence was computed dividing the estimated annual number of cases by the census population. We estimated the uncertainty bounds based on the 2.5th and 97.5th percentiles of the simulated distributions for the number of cases, from which we derived the ranges (from the minimum to the maximum value of such percentiles). All descriptive and statistical analyses were performed using Stata Statistical Software: Release 17 (StataCorp. 2023. College Station, TX: StataCorp LLC) [22].

### Ethical Approval

We used aggregated deidentified records for this evaluation which was exempt from ethical review within the Pan American Health Organization (PAHO) and the countries. In addition, this analysis was reviewed by the Centers for Disease Control and Prevention (CDC) and conducted consistently with applicable federal law and CDC policy.

## RESULTS

### Influenza Surveillance in Study Population

**Figure 1.**
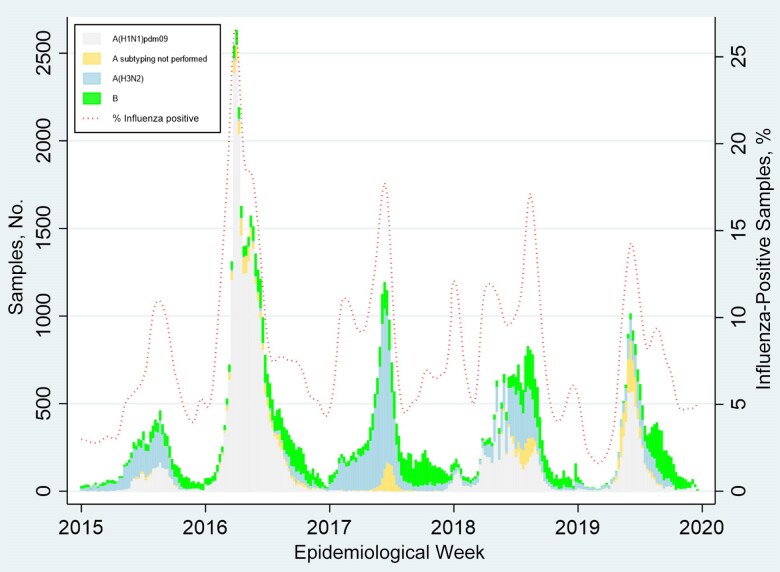
Influenza circulation by epidemiological week in Argentina, Brazil, Chile, Ecuador, Paraguay, and Uruguay, 2015–2019.

Participating countries had 307.4 million inhabitants, of whom 7.4% were <5, 83.0% were 5–64, and 9.6% were >64 years old (Table 1). During the study period, countries tested an annual average of from 668 (Uruguay) to 48 402 (Brazil) respiratory samples for influenza. Four of 6 countries provided virologic surveillance data only from inpatient samples, and 2 provided data from both inpatient and outpatient samples. The most commonly identified subtypes were influenza A/(H3) in 2015 and 2017 and influenza A/(H1N1)pdm09 in 2016, 2018, and 2019 (Figure 1 and Supplementary Table 1). The years of highest influenza B circulation were 2017 and 2019.

**Table 1.** Data Sources and Descriptions From Countries Contributing Data to the Estimate of Annual Influenza-Associated Respiratory Burden of Disease

Country	Influenza Transmission Zone	Total Population, Millions	Proportion of Population by Age Group, %			Source of Data			Diagnostics Used	Annual Value, Mean (SD)					
			<5 y	5–64 y	≥65 y	Hospital Discharge Codes	Mortality Data	Virologic Data		Respiratory Samples Tested, No.	Proportion of Samples Positive for Influenza, %	Respiratory Hospitalizations, No.	Proportion of Respiratory Hospitalizations	Deaths, No.	Proportion of Respiratory Deaths, %
Argentina^a^	Temperate South America	45.6	8.8	81.4	9.8	All hospitals; catchment area 100% of population	Directorate of Health Statistics and Information–Vital Statistics	Inpatient and outpatient specimens	RT-PCR and immunofluorescence assay	24 180 (4291)	5.4 (1.2)	782 389 (37 456)	11.2 (0.5)	92 299 (2207)	15.6 (0.5)
Brazil	Tropical South America	214.0	7.1	83.4	9.5	All federally supported hospitals; catchment area 75% of population; primary discharge diagnoses considered	Mortality Information System	Inpatient specimens	RT-PCR and immunofluorescence assay; 10% of negative samples by immunofluorescence assay are retested with RT-PCR	48 402 (19 500)	16.5 (3.9)	12 901 422 (402 354)	18.5 (0.1)	1 307 715 (30 881)	11.9 (0.1)
Chile	Temperate South America	19.2	6.3	81.9	11.8	All public and private hospitals; catchment area 100% of population primary discharge diagnosis considered	Department of Health Statistics and Information	Inpatient specimens	RT-PCR and immunofluorescence assay	5314 (493)	12.4 (1.4)	1 656 450 (17 621)	9.0 (0.8)	104 415 (5422)	10.1 (0.5)
Ecuador	Tropical South America	17.9	9.6	83.1	7.3	Catchment area 100% of population	National Institute of Statistics and Census	Inpatient specimens	RT-PCR	3945 (645)	10.2 (5.5)	1 158 557 (25 232)	7.3 (0.4)	70 028 (3437)	10.3 (0.4)
Paraguay	Temperate South America	7.2	9.9	83.6	6.5	All public and social security hospitals; catchment area 89% of population primary discharge diagnosis considered	General Directorate of Strategic Health Information	Inpatient specimens	RT-PCR and immunofluorescence assay	6321 (1467)	8.7 (1.3)	251 068 (65 763)	10.8 (0.7)	30 486 (1611)	9.1 (1.0)
Uruguay	Temperate South America	3.5	6.5	79.1	14.4	All hospitals; catchment area 100% of population	Ministry of Health–Vital Statistics	Inpatient and outpatient specimens	RT-PCR	668 (204)	13.3 (4.2)	360 430 (7099)	11.4 (0.3)	33 870 (776)	10.3 (0.5)

Abbreviation: RT-PCR, reverse-transcription polymerase chain reaction.

^a^For Argentina, data are from 16 jurisdictions.

### Respiratory Hospitalizations, Attributable Fraction of Influenza, and Associated Incidence

**Figure 2.**
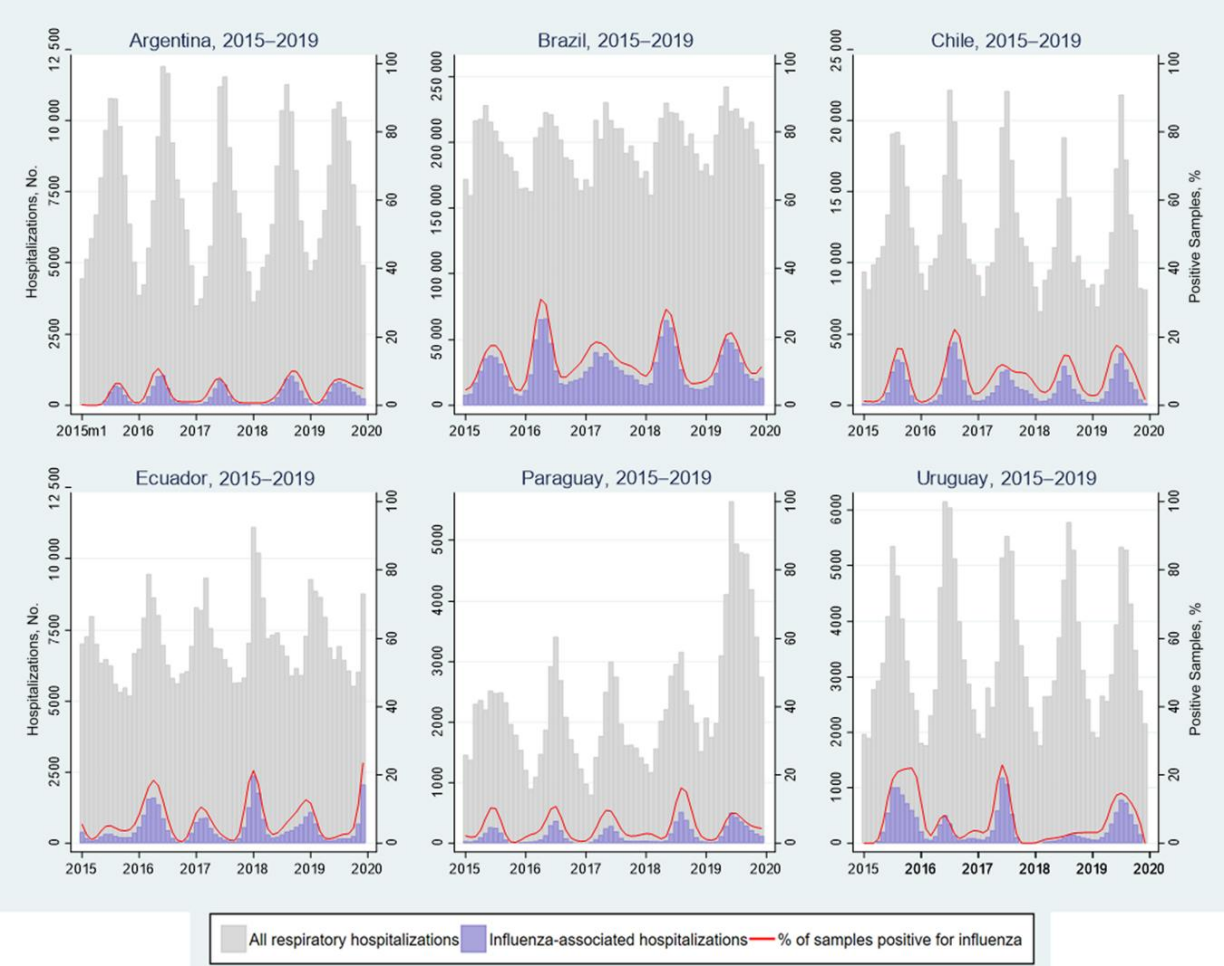
All respiratory and influenza-associated hospitalizations by month and year and across all age groups in Argentina, Brazil, Chile, Ecuador, Paraguay, and Uruguay, 2015–2019.

The absolute number of respiratory hospitalizations varied substantially by country, age group, and year (Table 2). During 2015–2019, the lowest incidences of respiratory hospitalizations in children <5 years old were in Ecuador, Brazil, and Paraguay, whereas the highest were in Chile and Uruguay. In people aged 5–64 years, incidence rates were highest in Brazil and lowest in Paraguay. Among adults aged ≥65 years, the incidence of respiratory hospitalizations was similar in most countries. Influenza-associated respiratory hospitalizations among children <5 years old ranged from 219–789 (minimum value of the 2.5th percentile to maximum value of the 97.5th percentile) in Paraguay to 13 505–36 421 in populous Brazil (Table 2 and Figure 2). Among adults aged ≥65 years, influenza-associated respiratory hospitalizations also ranged from 350–853 in Paraguay to 37 046–85 815 in Brazil. Consequently, the lowest number of influenza hospitalizations among all age groups was in Paraguay (ie, 1084–3387) and the highest in Brazil (285 309–434 327). The annual incidence of influenza-associated hospitalizations ranged from 16–47/100 000 in Paraguay to 140–208/100 000 in Brazil. During 2015–2019 across all countries, the estimated number of influenza-associated hospitalizations ranged from 323 379 to 490 049 and varied substantially by country, age group, and year (Figure 4 and Supplementary Tables 2–4). For example, the number of influenza-associated hospitalizations ranged from 323 379–345 936 in 2015 to 465 526–490 049 in 2018.

**Figure 3.**
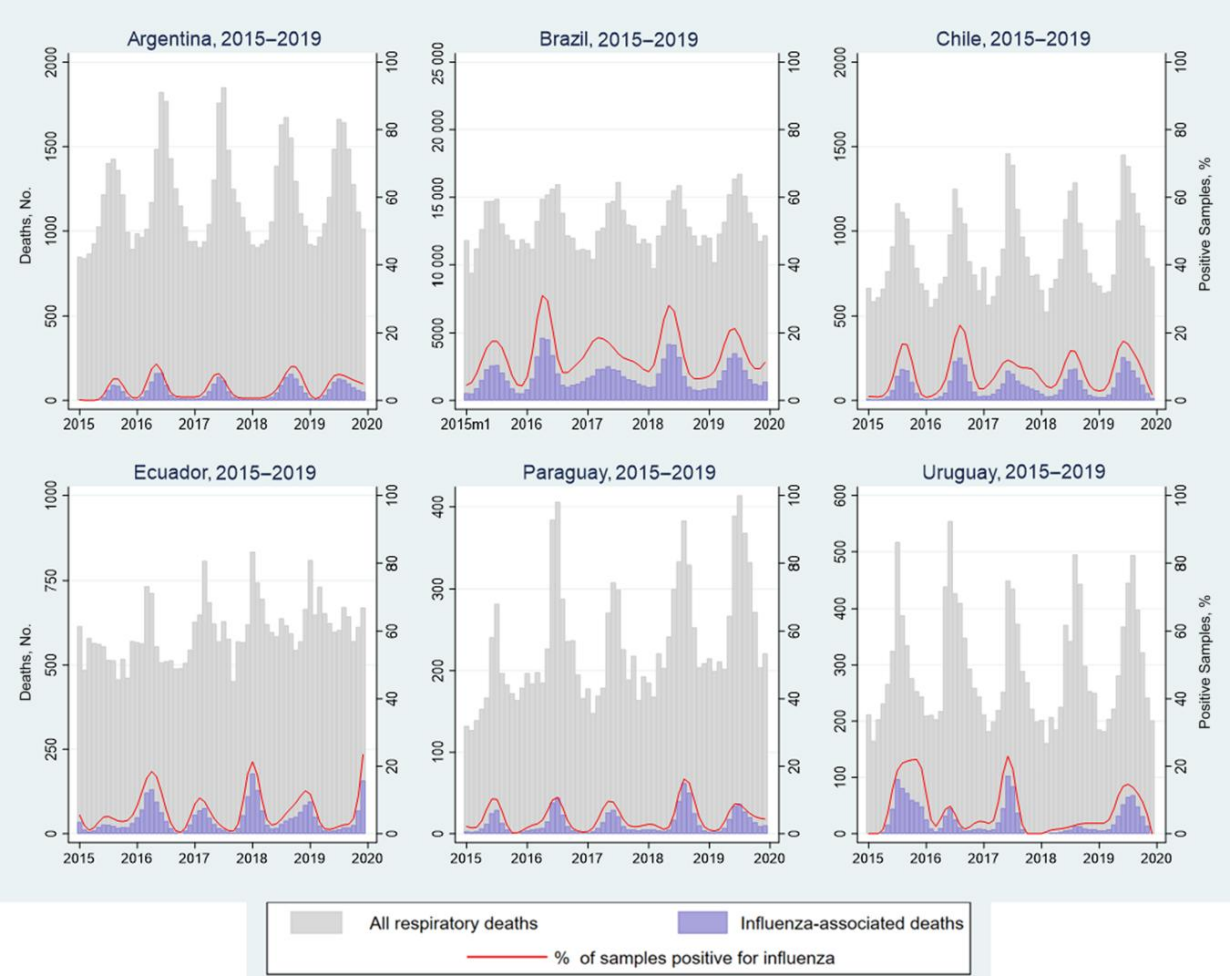
All respiratory and influenza-associated deaths by month and year and across all age groups in Argentina, Brazil, Chile, Ecuador, Paraguay, and Uruguay, 2015–2019.

**Figure 4.**
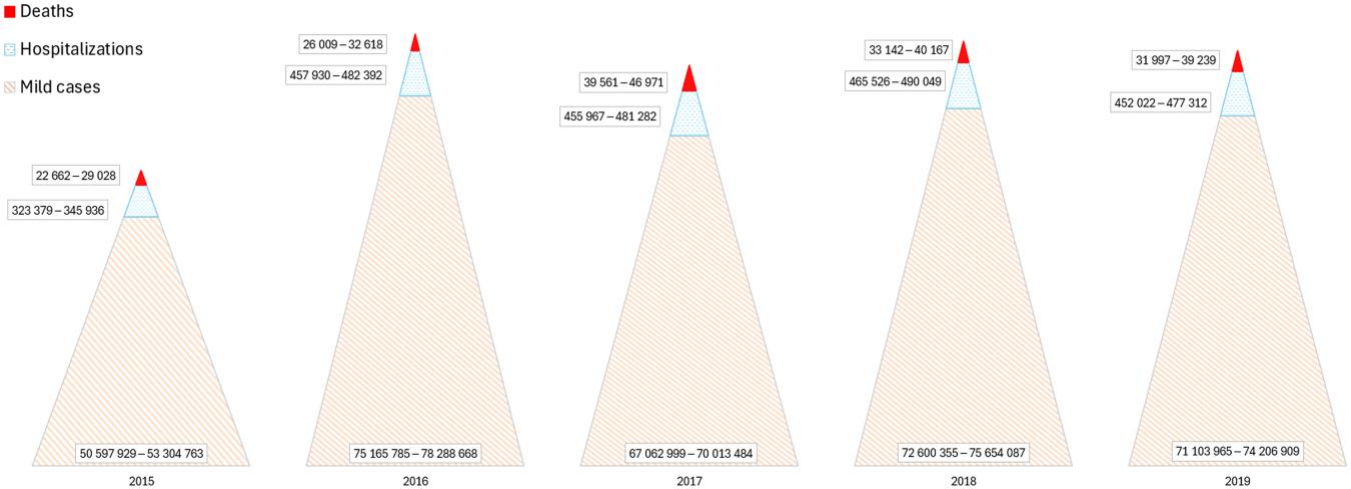
Estimated influenza burden of disease by year and across all age groups in Argentina, Brazil, Chile, Ecuador, Paraguay, and Uruguay, 2015–2019. The area inside the pyramid represents the total number of cases (midpoint of the range) of each type. Ranges were obtained from the minimum value of the 2.5th percentile and the maximum value of the 97.5th percentile in the Monte Carlo simulations.

**Table 2.** Ranges of All Annual Respiratory Hospitalizations and Estimated Influenza-Associated Hospitalizations by Age Group in Argentina, Brazil, Chile, Ecuador, Paraguay, and Uruguay, 2015–2019

Measure by Country	All Respiratory Hospitalizations (Range^a^)				Influenza-Associated Hospitalizations (Range^b^)			
	Age <5 y	Age 5–64 y	Age ≥65 y	All Ages	Age <5 y	Age 5–64 y	Age ≥65 y	All Ages
No. of hospitalizations								
Argentina^c^	36 858–41 978	28 244–32 159	16 150–17 084	81 822–90 586	1385–4615	6714–15 055	4485–10 517	16 138–28 228
Brazil	340 074–365 551	1 618 258–1 773 529	345 616–378 349	2 313 334–2 506 780	13 505–36 421	232 694–347 647	37 046–85 815	285 309–434 327
Chile	46 301–50 381	45 617–60 875	35 428–51 417	129 610–157 754	1400–3376	4199–9267	4730–9758	10 536–20 160
Ecuador	28 918–33 522	30 524–35 334	17 201–21 837	76 643–90 693	601–2648	1267–6634	857–3952	2725–13 234
Paraguay	9873–21 254	6521–13 097	4897–9172	21 432–43 523	219–789	472–1127	350–853	1084–3387
Uruguay	11 822–14 532	13 684–14 730	12 947–14 107	39 466–43 183	202–2128	188–2052	195–2135	585–6315
Incidence per 100 000 population								
Argentina^c^	2967–3386	250–291	1168–1401	592–671	37–124	19–42	94–227	37–63
Brazil	2311–2491	943–1011	1944–2046	1128–1193	92–248	136–201	208–464	140–208
Chile	3787–4059	297–407	1567–2483	691–878	116–274	27–62	228–471	56–106
Ecuador	1720–2012	226–250	1551–1788	471–533	36–159	9–47	77–324	17–78
Paraguay	1406–3013	112–219	1139–1964	308–608	31–112	8–19	81–190	16–47
Uruguay	5195–6319	497–533	2673–2853	1122–1241	89–921	7–75	39–441	17–182

^a^Ranges were obtained from minimum and maximum values of the series.

^b^Ranges were obtained from the minimum value of the 2.5th percentile and the maximum value of the 97.5th percentile in the Monte Carlo simulations.

^c^For Argentina, input data were available from 16 jurisdictions, but influenza estimates were extrapolated to the whole country.

### Respiratory Deaths, Attributable Fraction of Influenza, and Associated Incidence

During 2015–2019, the absolute number and incidence of respiratory deaths per year in older adults (aged ≥65 years) were higher than in the other age groups for all countries (Table 3 and Figure 3). Among children aged <5 years, the numbers of influenza-associated respiratory deaths ranged from 0–17 in Chile to 43–403 in populous Brazil. Among adults aged ≥65 years, the numbers of influenza-associated respiratory deaths also ranged from 25–565 in Uruguay to 12 143–27 970 in Brazil. Consequently, the lowest number of influenza deaths among all age groups was in Paraguay (ie, 109–521), and the highest in Brazil (16 306–34 444). The annual incidence of influenza-associated deaths ranged from 2–7/100 000 in Paraguay to 9–19/100 000 in Argentina.

**Table 3.** Ranges of All Annual Respiratory Deaths and Estimated Influenza-Associated Deaths by Age Group in Argentina, Brazil, Chile, Ecuador, Paraguay, and Uruguay, 2015–2019

Measure by Country	All Respiratory Deaths (Range^a^)				Influenza-Associated Deaths (Range^b^)			
	Age <5 y	Age 5–64 y	Age ≥65 y	All Ages	Age <5 y	Age 5–64 y	Age ≥65 y	All Ages
No. of deaths								
Argentina^c^	181–276	2010–2513	10 797–12 447	13 013–15 002	0–85	270–1350	3268–7384	3918–8651
Brazil	2349–2689	30 291–34 869	116 084–127 087	149 333–161 884	43–403	4120–8270	12 143–27 970	16 306–34 444
Chile	39–58	1247–1699	8539–9837	9847–11 588	0–17	84–354	747–1935	852–2204
Ecuador	310–357	1143–1493	4936–6139	6395–7833	0–62	23–377	209–1128	232–1567
Paraguay	106–160	427–665	1573–2499	2136–3294	0–30	9–118	100–383	109–521
Uruguay	10–18	392–488	2897–3310	3302–3816	0–18	0–103	25–565	25–686
Incidence per 100 000 population								
Argentina^c^	15–22	18–23	885–968	96–110	0–2	1–4	69–145	9–19
Brazil	16–18	17–20	635–678	73–77	0–3	2–5	71–151	8–17
Chile	3–5	8–11	412–459	54–61	0–1	1–2	39–93	5–12
Ecuador	18–21	8–11	445–486	39–45	0–4	0–3	19–92	1–9
Paraguay	15–23	8–11	396–535	32–46	0–4	0–2	25–86	2–7
Uruguay	4–8	14–18	586–677	95–110	0–8	0–4	5–117	1–20

^a^Ranges were obtained from minimum and maximum values of the series.

^b^Ranges were obtained from the minimum value of the 2.5th percentile and the maximum value of the 97.5th percentile in the Monte Carlo simulations.

^c^For Argentina, input data were available from 16 jurisdictions, but influenza estimates were extrapolated to the whole country.

During 2015–2019 across all countries, the estimated number of influenza-associated deaths ranged from 22 662 to 46 971 (minimum to maximum value of the series) and also varied substantially by country, age group, and year (Figure 4 and Supplementary Tables 2–4). For example, the number of influenza-associated deaths ranged from 22 662–29 028 (minimum value of the 2.5th percentile to maximum value of the 97.5th percentile) in 2015 to 39 561–46 971 in 2018.

### Mild-to-Moderate Influenza Illness Estimates Across All Countries

During 2015 to 2019, the estimated number of mild-to-moderate influenza illnesses ranged from 50.6 to 78.3 million (Figure 4 and Supplementary Tables 2–4). This represents an influenza illness attack rate of 16.5%–25.5% among 307 million people in Argentina, Brazil, Chile, Ecuador, Paraguay, and Uruguay. This number of mild-to-moderate influenza cases ranged from 50.6–53.3 million cases in 2015 to 75.2–78.3 million cases in 2016.

## DISCUSSION

Our estimates suggest that vaccine-preventable viral influenza caused substantial disease and death every year in Argentina, Brazil, Chile, Ecuador, Paraguay, and Uruguay. During 2015–2019, influenza sickened >1 in 6 persons each epidemic period. Our estimated influenza hospitalizations and deaths were also similar to those of the United States, which has a population of 336 million, similar to that of Argentina, Brazil, Chile, Ecuador, Paraguay, and Uruguay combined (ie, 307 million people). During 2015–2019 the United States had similar influenza hospitalizations (ie, 270 000–710 000 vs our 323 000–490 000) and deaths (ie, 22 000–51 000 vs our 23 000–47 000) [23]. Only the US mild-to-moderate disease estimates seemed lower than those from our study countries (ie, 23–41 million vs our 51–78 million cases [23]). Our findings were also similar to previous influenza-associated hospitalizations estimates [7] and deaths [8] from Latin America.

In Latin America and the Caribbean, few studies have estimated the burden of mild-to-moderate disease. In a systematic review and meta-analysis by Savy et al [24], only 4 studies estimated rates of influenzalike illness. Those findings are not comparable to ours because they are derived using different methods to define mild illness. Estimating the complete spectrum severity is challenging, because few persons are tested for influenza, and the majority of those ill remain undiagnosed [25, 26]. Furthermore, influenza illness may present as nonrespiratory illness, especially among the extremes of age [27]. The inclusion of these additional outcomes would increase the BoD. PAHO and WHO are working with academic partners to improve analytic methods and better estimate the burden of nonrespiratory, non–medically attended influenza illness [28]. Such efforts are anticipated to help estimate the cost-benefit of influenza vaccination among target groups.

Our results also demonstrate how there is year-to-year variability in the influenza-associated burden. Several factors contribute to this variability, such as prior immunity among persons of different age, the predominance of circulating virus types and subtypes, the antigenic similarity between circulating viruses and vaccines, and vaccination coverage [29, 30]. Our evaluation demonstrates that South American countries can use surveillance, hospital records, and vital statistics to estimate the interim, or end-of-season, influenza burden as is commonly done in other countries each season [6]. Interim estimates help health authorities differentiate mild versus severe influenza seasons, guide risk communications, serve as inputs for vaccine averted illness models, and inform populations about the benefit of influenza vaccines and antivirals. End-of-season estimates can also be used as inputs for cost-effectiveness analyses, when countries are exploring an investment case for procuring vaccines and antivirals, guide next influenza season's procurement, and inform epidemic and pandemic preparedness planning (eg, when estimating hospital staff and bed surge capacity requirements) [31].

SARInet facilitates the exchange of surveillance information, helps countries estimate respiratory virus incidence, guides regional health policies, and helps mitigate influenza. In addition to SARInet, the Network for the Evaluation of Vaccine Effectiveness in Latin America and the Caribbean (REVELAC-i) estimates interim and end-of-season influenza vaccine effectiveness using influenza surveillance and vaccine registry data [32]. In 2019, for example, REVELAC-i demonstrated that countries could prevent more than a third of influenza-associated hospitalizations in young children and older adults by vaccinating everyone in these target groups [33]. SARInet BoD findings coupled with REVELAC-i vaccine effectiveness findings can also serve as inputs for vaccine averted illness models, which help inform providers and target groups about the benefits of influenza vaccination.

Our study has several limitations. First, we used 2 multiplier methods to estimate the burden of severe disease and mild-to-moderate illness. Multiplier methods can generate underestimates because, for example, they rely on specific case definitions (eg, influenza-associated respiratory hospitalizations) rather that incorporating all manifestation of influenza illness (eg, influenza-associated respiratory and circulatory hospitalizations) [34, 35]. Work is underway to refine and validate a novel multiplier method for future influenza BoD estimates, as our methodologic approach to mild disease was not yet validated and relied on data published in peer-reviewed literature, with data from other regions. Second, we could not properly adjust for the possible correlation between the data sets. For this reason, we presented the results in ranges, rather than confidence intervals, to increase the amplitude of the interval, so that the true value may be contained in our estimates. Third, our results may underestimate influenza-associated hospitalizations because national discharge datasets comprised mostly data from public hospitals and may have missed data from some private hospitals, especially in Paraguay; our hypothesis, however, is that this effect is not very high, since about 27% of the population is served in the private system, according to the National Institute of Statistics of Paraguay. Finally, testing schedules and regimens might vary between countries and therefore might have influenced the positivity in the different health care levels. Moreover, the different healthcare systems in the countries and related surveillance system set-ups might may an impact on the respective country data, and care seeking likely varied from year to year; however, we assumed that care seeking at hospitals was similar from year to year.

In conclusion, each year between 2015 and 2019, influenza caused millions of illnesses, hundreds of thousands of hospitalizations, and tens of thousands of deaths in 6 of the 12 countries in South America. Indeed, between 1 in 6 and a quarter of the population could become sick from influenza annually. Such findings are useful for decision makers. Awareness of influenza BoD and severity has been identified as one of the 5 pillars that support successful influenza vaccination programs and can inform future policies aiming to achieve and maintain better influenza control [36]. Influenza BoD data can be used to identify and target undervaccinated populations; communicate influenza risks to the public; guide research funding to areas with the greatest potential impact, such as new vaccines and antiviral drugs; guide epidemic and pandemic planning; and assess the cost-effectiveness of interventions.

## Supplementary Data

Supplementary materials are available at *The Journal of Infectious Diseases* online (http://jid.oxfordjournals.org/). Supplementary materials consist of data provided by the author that are published to benefit the reader. The posted materials are not copyedited. The contents of all supplementary data are the sole responsibility of the authors. Questions or messages regarding errors should be addressed to the author.

## Supplementary Material

jiaf037_Supplementary_Data

## Notes

***Acknowledgments*.** We thank Olaya Astudillo for reviewing, editing and input, and we acknowledge the contributions of SARInet working groups, the PAHO country focal points, and the ministries of health staff in Argentina, Brazil, Chile, Ecuador Paraguay, and Uruguay. We also thank all colleagues that have been part of the influenza team at PAHO for their collaboration through this 10-year journey, especially Rakhee Palekar, former coordinator of the team.

***Author contributions*.** Conceptualization and supervision: M. A. D., J. J., A. R., S. T., and P. C. Data curation and validation: M. A. D., F. J. d. P. J., N. V. M., E. P., C. Voto, N. G., A. B., W. A. F. d. A., G. M. I. d. C., M. F. O. B., R. F., J. P., C. Vázquez, M. V. H., S. B., C. G., E. B., A. S., M. P. B., M. A., M. P. E. N., J. J., and P. C. Formal analysis and software: M. A. D. Methodology: M. A. D., J. J., S. T., and P. C. Project administration: M. A. D., A. R., and P. C. Visualization: M. A. D., J. J., N. J. A. Z., and P. C. Writing–original draft preparation: M. A. D. and P. C. Writing–review and editing: All authors. M. A. D. had full access to all the data in the study and takes responsibility for the integrity of the data and the accuracy of the data analysis. All authors have reviewed and approved the final version of the manuscript.

***Data availability*.** All relevant data are provided in the paper and its supporting information files.

***Financial support*.** This work was supported by the Centers for Disease Control and Prevention and PAHO (cooperative agreement NU51IP000940) and the World Health Organization (Pandemic Influenza Preparedness Framework).

***Supplement sponsorship.*** This article appears as part of the supplement “Advancing Respiratory Virus Surveillance and Preparedness in the Americas: A Decade of SARInet's Impact,” sponsored by the Pan American Health Organization.

***Potential conflicts of interest*.** All authors: No reported conflicts. All authors have submitted the ICMJE Form for Disclosure of Potential Conflicts of Interest. Conflicts that the editors consider relevant to the content of the manuscript have been disclosed.
