# Supplementary material for: Annual Estimation of Seasonal Influenza Burden in 6 South American Countries: A Retrospective Analysis of SARInet Surveillance Data to Inform Policies
Source: J Infect Dis. 2025 Mar 10;231(Suppl 2):S123–32. doi: 10.1093/infdis/jiaf037 (PMC11892003; doi:10.1093/infdis/jiaf037)
Supplement: jiaf037_Supplementary_Data [file jiaf037_supplementary_data.docx]

Supplementary material: Annual estimation of seasonal influenza in SARInet

**Supplementary Table 1.- Summary of predominant* circulating influenza virus among contributing countries by year (2015 — 2019) in percentages.**

| **Year** | **A(H1N1)pdm09** | **A subtyping not performed** | **A(H3N2)** | **B Victoria** | **B Yamagata** | **B not determined** |
| --- | --- | --- | --- | --- | --- | --- |
| **2015** | 25 | 2 | **51** | 4 | 5 | 13 |
| **2016** | **79** | 5 | 3 | 3 | 1 | 10 |
| **2017** | 1 | 6 | **62** | 1 | 8 | 21 |
| **2018** | **41** | 6 | **30** | 2 | 7 | 14 |
| **2019** | **42** | 13 | 19 | 1 | 9 | 16 |

*We classified the predominant virus based on the percentage of each type/subtype (number of positive samples of each sub type/subtype among the total annual influenza-positive samples sent to the national virological surveillance).

Percentage of the annual influenza-positive specimens between ≥30% and ≤50% are shown in light green

Percentage of the annual influenza-positive specimens >50% are shown in light blue

**Supplementary Table 2.- Estimated Influenza Burden of Disease in children younger than 5 years by year in Argentina, Brazil, Chile, Ecuador, Paraguay, and Uruguay, 2015–2019.**

| **Age group** | **Year** | **Influenza-associated deaths (range‡)** | **Influenza-associated hospitalizations (range‡)** | **Influenza-associated mild-to-moderate cases (range‡)** |
| --- | --- | --- | --- | --- |
| **Aged <5 years** | **2015** | 43–322 | 19,165–23,326 | 2,242,305–2,729,142 |
|  | **2016** | 193–572 | 41,538–46,857 | 4,859,946–5,482,269 |
|  | **2017** | 69–367 | 25,468–30,065 | 2,979,756–3,517,605 |
|  | **2018** | 139–473 | 35,101–40,054 | 4,106,817–4,686,318 |
|  | **2019** | 124–479 | 35,673–41,093 | 4,173,741–4,807,881 |

‡ Ranges were obtained from the minimum value of the 2.5th percentile and the maximum value of the 97.5th percentile of the Monte Carlo simulations.

**Supplementary Table 3.- Estimated Influenza Burden of Disease in adults aged 5–64 years by year in Argentina, Brazil, Chile, Ecuador, Paraguay, and Uruguay, 2015–2019.**

| **Age group** | **Year** | **Influenza-associated deaths (range‡)** | **Influenza-associated hospitalizations (range‡)** | **Influenza-associated mild-to-moderate cases (range‡)** |
| --- | --- | --- | --- | --- |
| **Aged 5–64 years** | **2015** | 4,542–6,242 | 249,502–260,497 | 47,425,520–49,519,700 |
|  | **2016** | 8,158–10,435 | 365,426–377,974 | 69,440,250–71,829,120 |
|  | **2017** | 5,899–7,940 | 328,126–340,021 | 62,342,800–64,605,700 |
|  | **2018** | 6,867–9,045 | 353,371–365,718 | 67,183,620–69,535,060 |
|  | **2019** | 6,471–8,561 | 345,874–358,171 | 65,733,730–68,074,150 |

‡ Ranges were obtained from the minimum value of the 2.5th percentile and the maximum value of the 97.5th percentile of the Monte Carlo simulations.

**Supplementary Table 4.- Estimated Influenza Burden of Disease in adults older than 64 years by year in Argentina, Brazil, Chile, Ecuador, Paraguay, and Uruguay, 2015–2019.**

| **Age group** | **Year** | **Influenza-associated deaths (range‡)** | **Influenza-associated hospitalizations (range‡)** | **Influenza-associated mild-to-moderate cases (range‡)** |
| --- | --- | --- | --- | --- |
| **Aged ≥65 years** | **2015** | 18,077–22,464 | 54,712–62,113 | 930,104–1,055,921 |
|  | **2016** | 17,658–21,611 | 50,917–57,487 | 865,589–977,279 |
|  | **2017** | 33,593–38,664 | 102,379–111,187 | 1,740,443–1,890,179 |
|  | **2018** | 26,136–30,649 | 77,054–84,277 | 1,309,918–1,432,709 |
|  | **2019** | 25,402–30,199 | 70,382–77,934 | 1,196,494–1,324,878 |

‡ Ranges were obtained from the minimum value of the 2.5th percentile and the maximum value of the 97.5th percentile of the Monte Carlo simulations.
